# Cytological diagnosis of thyroid nodules in Hashimoto thyroiditis in elderly patients

**DOI:** 10.1186/1471-2482-13-S2-S41

**Published:** 2013-10-08

**Authors:** Alessia Caleo, Elena Vigliar, Mario Vitale, Vincenzo Di Crescenzo, Mariapia Cinelli, Chiara Carlomagno, Alfredo Garzi, Pio Zeppa

**Affiliations:** 1 Department of Medicine and Surgery, University of Salerno, Italy; 2 Departments of Biomorphological and Functional Sciences, University of Naples "Federico II", Italy; 3 Clinical Medicine and Surgery, University of Naples "Federico II" Italy; 4 Public Health, University of Naples "Federico II", Italy

## Abstract

**Background:**

Long standing Hashimoto Thyroiditis (HT) causes shrinking and atrophy of the thyroid, but may also lead to diffuse enlargement of the gland and/or formation of nodules. These nodules should be differentiated from papillary thyroid carcinoma (PTC) and primary thyroidal non-Hodgkin lymphoma (PTL), which are possible complications of HT, and require pre-surgical diagnoses and different treatments.

This study focuses on the role of fine-needle cytology (FNC) in the clinical surveillance and pre-surgical diagnosis of HT with diffuse and nodular enlargement of the gland in elderly patients.

**Methods:**

Thirty-four elderly patients (≥ 65 yrs) with HT and diffuse or nodular enlargement of the thyroid underwent ultrasound (US)-guided FNC. Smears were routinely stained and evaluated; additional passes were used for flow cytometry (FC) assessment of lymphoid infiltrate in 6 cases.

**Results:**

The cytological diagnosis was HT in 12 cases with prevalence of Hurtle cells in 2 cases, PTC in 1 case and PTL in 2 cases. FC assessed the reactive, non-lymphomatous nature of the lymphoid infiltrate in 5 cases and demonstrated light chain restriction, hence the lymphomatous nature of the lymphoid infiltrate in 2 cases of PTL.

**Conclusions:**

FNC plays a key role in the clinical surveillance and pre-surgical diagnosis of diffuse enlargement and nodular presentation of HT in elderly patients. FNC can correctly diagnose HT, PTC and PTL indicating the need for surgery and its extension in suspicious or neoplastic cases, leaving other cases to the medical treatment and clinical surveillance.

## Introduction

Hashimoto Thyroiditis (HT) is the most common autoimmune disease; the typical clinical presentation is a painless diffuse enlargement of the thyroid, with high serum thyroid autoantibodies [1,2]. HT is characterized by the progressive loss of follicular cells and their concomitant replacement by a lymphoid infiltrate (TLI) with the formation of germinal centers and fibrosis[1-3]. HT patients have high levels of serum thyroid autoantibodies which include antithyroid peroxidase (anti-TPO), antithyroglobulin (anti-Tg), whereas a percentage of patients (10-15%) may be antibody negative. An increase in the serum thyroid stimulating hormone (TSH), in any phase of the disease, heralds incipient hypothyroidism [3]. Lymphoid infiltrate in HT release in situ a number of factors, including cytokines, that increase the intracellular generation of reactive oxygen species (ROS). ROS have important cellular and tissue effects and are responsible for senescence, cancer and other age-related diseases [4-6]. Over time, the gland progressively shrinks causing almost complete atrophy but, in many patients and/or in different phases of the disease, there is a symmetrical or asymmetrical enlargement of the gland with nodule formation [7,8]. Indeed, US presentation of HT varies significantly, ranging from a normal thyroid parenchyma imaging to diffuse homogeneous enlargement or atrophy, up to uninodular or multinodular presentation in +/-50% of cases [7,8]. Nodule US appearance is also extremely variable: nodules may be hypo or hyper echoic, can have calcifications (micro and scattered, macro or eggshell calcifications), and may also show a hypoechoic halo [7,8]. With reference to the clinical behavior, possible HT complications include papillary carcinoma (PTC) [9-11] and, rarely, primary non-Hodgkin lymphoma (PTL) [3,12-14], in addition to hypothyroidism [1,15,16]; therefore a direct evaluation of diffuse or nodular thyroid enlargement in case of HT may be needed. The clinical appearance of thyroid cancer is that of a nodules, some time representing a challenging diagnostic dilemma with thyroid or unusual extrathyroidal masses [17,18]. Fine-needle cytology (FNC) is widely used in the diagnosis of thyroid nodules [18-20], and the application of molecular techniques to FNC has dramatically increased its sensitivity [3,21-29], including in cases of HT with diffuse or nodular enlargement [3]. An effective FNC diagnosis avoids useless diagnostic surgery or leads to the proper surgical treatment, when needed [30][18]. These advantages are enhanced in the case of HT which does not require surgical treatment, especially in elderly patients in which any surgery is generally more burdensome, complex and expensive than younger patients [31,32]. The aim of this study is to assess the role of FNC in the clinical surveillance and pre-surgical diagnosis of thyroidal diffuse enlargement and HT nodules in elderly patients.

## Materials and methods

Between January 2010 and December 2012, 1.256 patients with thyroidal nodules or diffuse swelling underwent FNC in the outpatient clinics of the Azienda Ospedaliera Universitaria, University of Salerno. Before FNC, patients were informed of the diagnostic procedure and related risks; informed consent was obtained from all patients for the performance of FNC, diagnostic procedures and the scientific use of biological material. Sixty patients were diagnosed with HT or suspected HT because of the clinical, serologic, and/or ultrasound (US) presentation. Clinical signs and symptoms included swollen thyroid gland, with or without discrete nodules and, rarely, mild fever. US examination generally showed poorly defined hypoechoic areas, thin fibrous strands with diffuse hypervascular pattern or diffuse glandular enlargement with scattered nodules. Clinical, serological and US data were available for all patients and FNC produced a sufficient amount of cells to prepare adequate cytological smears and perform ancillary techniques, when needed. Whereas the definition of elderly is related to the pathophysiology of aging [31-33], the criteria of eligibility in the present study considered elderly patients those older than 65 years [34], therefore we enrolled 14 patients (13 women and 1 man; age range 65-73 years). All patients showed palpable nodular or diffuse enlargement of the thyroid, some of them received previous pharmacologic treatments for thyroid pathologies and, according to the US pattern, they were classified as "nodular" in 12 cases and "diffuse" in 2 cases. Serologic assessment for TSH, free triiodothyronine (FT3), free thyroxine (FT4), thyroglobulin antibodies and thyroid peroxidase antibodies (TPO-AB) was performed in all cases. The median values were as follows: TSH: 2.8 U/L (range, 0.9- 16.1 U/L); free triiodothyronine (FT3): 3.4 pg/mL (range, 2.9-4.0 pg/mL); free thyroxine (FT4): 12.1 pg/mL (range, 8.0-14.3 pg/mL); thyroglobulin antibodies (Tg-AB): 212 U/L (range, 86-1240 U/L); and thyroid peroxidase antibodies (TPO-AB): 89 (range, 48-612 U/L).

### *Fine-needle cytology*

The diagnostic procedure and related risks were first discussed with the patients, who were also informed that 1 or 2 supplementary passes might have been needed, and their informed consent was obtained. FNC was performed under US control, as previously described [35,36], and 2 or more smears were prepared according to the amount of material obtained. In 20 cases, a rapid on site evaluation (ROSE) on Diff-Quik stained smears was performed as described elsewhere [36]. In 8 cases showing scanty cellular material, the procedure was repeated immediately or after a week, when ROSE was not performed. On the basis of the ROSE evaluation, a second pass was used in 6 cases to prepare additional smears and/or cell suspension for flow cytometry (FC) assessment of the lymphoid cell populations, as previously described [37-39]. According to the standard cytologic criteria [40], the diagnosis of HT was made on the basis of a lymphoid cell infiltrate, the presence of stretched lymphocytes, and multinucleated giant cells, if any. Follicular cells and Hurthle cells had to be present in variable numbers and were re-evaluated according to the current diagnostic criteria. FC data were interpreted accordingly [3] in this specific clinical and anatomical setting. With regard to the light chain evaluation for clonal assessment of lymphoid cell populations, a percentage of the gated cells showing κ/λ unbalance ≤ 20% of the gated cells was considered as an evidence of clonality [3,41]. FC assessment was used to perform the final diagnosis and was included in the cytological reports. All the cytological diagnoses were confirmed by the clinical, serologic, and US follow-up in case of HT and by histological examination in 2 cases cytologically diagnosed as PTC (case 1) and PTL (case 2), respectively.

## Results

**Figure 1:**
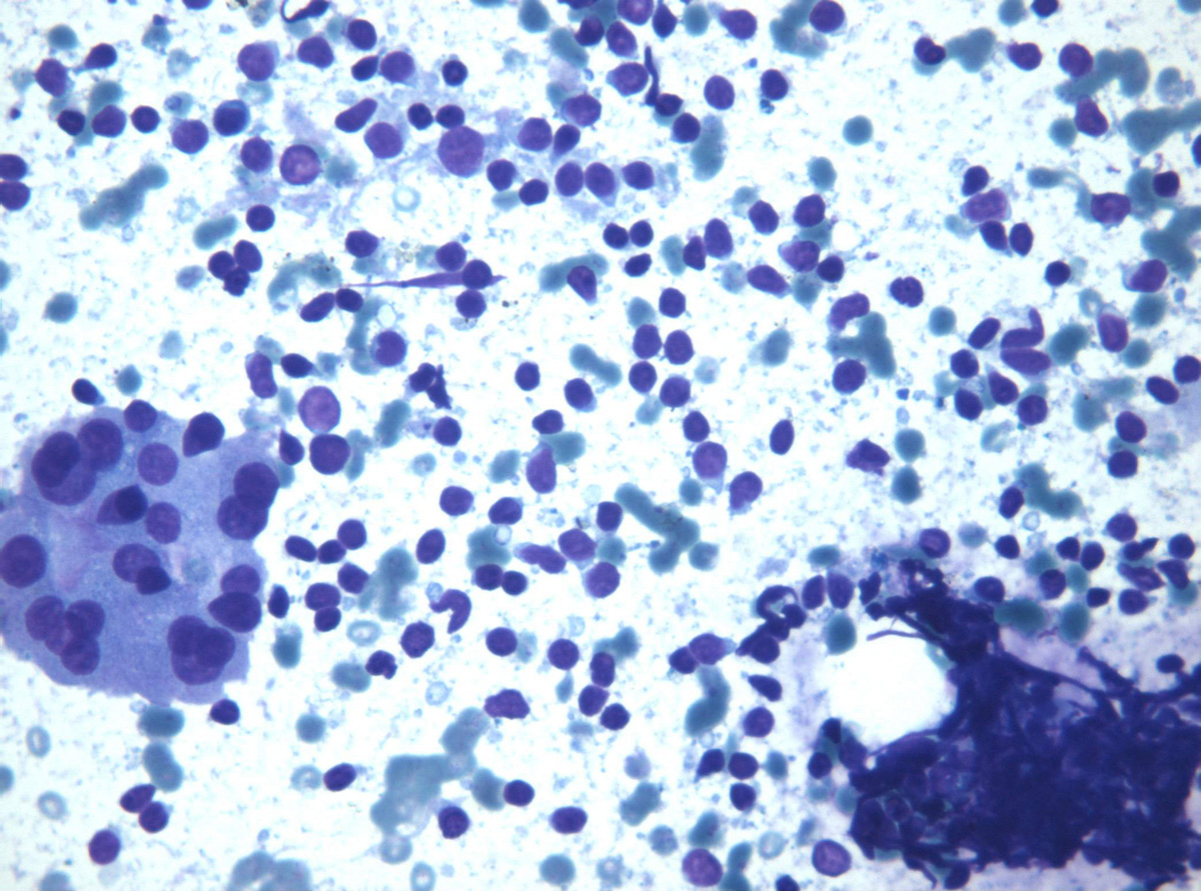
**Lymphoid cells were characterized by small lymphocytes, either isolated or intermingled with histiocytes, occasional plasma cells, and stretched lymphocytes; this pattern was referred as ''lymphocytic'' (Original magnification × 40)**.

**Figure 2:**
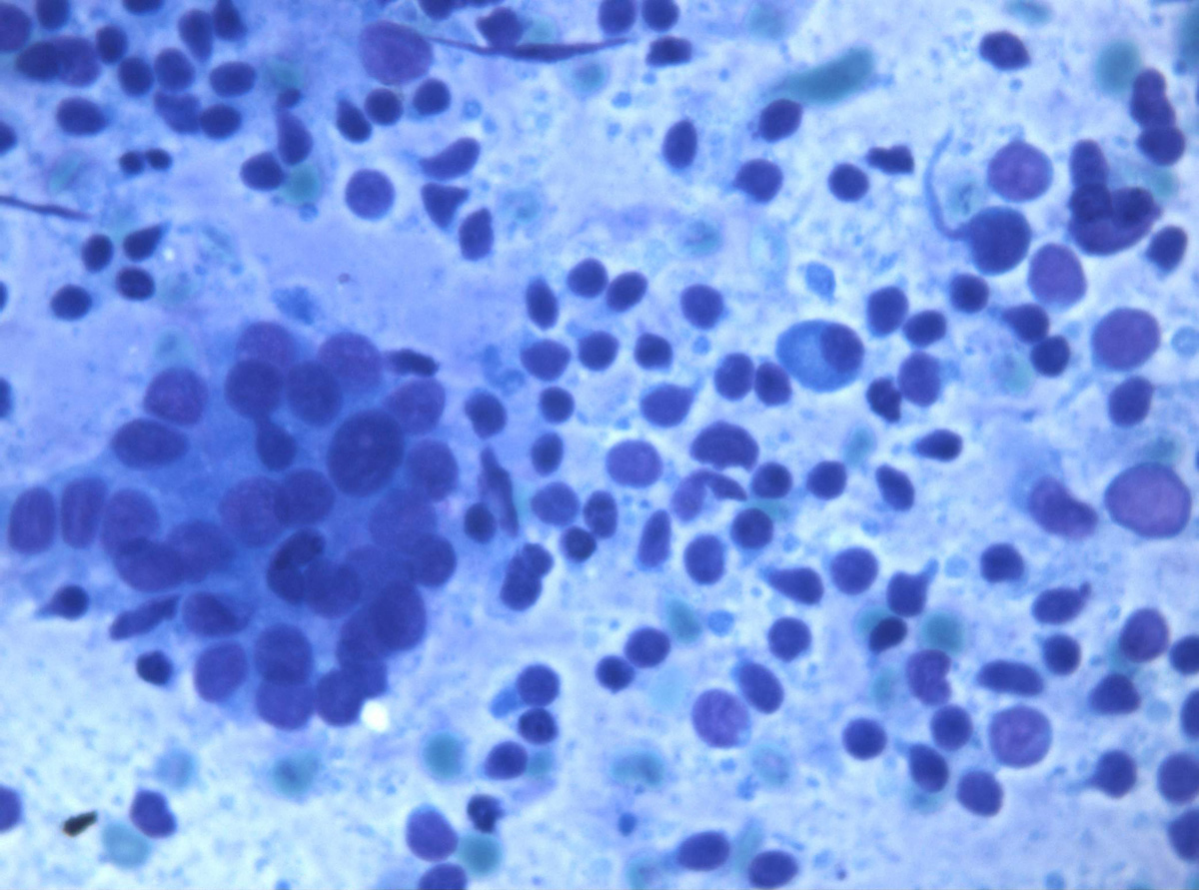
**Cells gave the smear a lymph node appearance, referred to as ''lymph node-like'' pattern (Original magnification × 40)**.

The cytological diagnosis was typical HT in 10 cases, HT with prevalence of Hurthle cells in 2 cases, PTC in 1 case and PTL in 1 case. Typical HT showed different cytological patterns due to the prevalence of lymphoid cells or follicular and Hurthle cells. Those with prevalence of lymphoid cells were characterized by small lymphocytes, either isolated or intermingled with histiocytes, occasional plasma cells, and stretched lymphocytes. This pattern was referred as ''lymphocytic'' (6 cases) (Figure 1). In addition to the small lymphocyte and plasma cell population, a second cell population was observed, having larger lymphocytes and germinal center lymphoid cells. These cells gave the smear a lymph node appearance, referred to as ''lymph node-like'' pattern (Figure 2) (4 cases). In these cases, a differential diagnosis with a possible Non-Hodgkin Lymphoma (NHL) was taken into account and FC data confirmed the reactive, non lymphomatous nature of the lymphoid infiltrate. Conversely, FC demonstrated the clonal lymphomatous nature of the lymphoid infiltrate in 2 PTL cases. In 2 cases, the prevalence of follicular cells and Hurthle cells was observed. In these cases a differential diagnosis with Hurthle cell neoplasm was taken into account; nonetheless the polymorphism of the cells, the concomitant presence of lymphoid infiltrate and stretched lymphocytes supported the diagnosis of HT. As for the neoplastic nodules, 2 cases were diagnosed as PTL and 1 case as PTC on the basis of cytological and FC data. PTL cases underwent systemic screening and positron emission tomography (PET) which assessed that thyroid was the only organ involved; therefore, patients underwent hemithyroidectomy. The histological examination confirmed the diagnosis of mucosa-associated lymphoid tissue (MALT) and diffuse large B-cell lymphoma (DLBCL). The patient who received the diagnosis of PTC underwent total thyroidectomy and radio metabolic therapy, being the cytological diagnosis confirmed by histology. All PTL and PTC patients are alive without signs of disease.

## Discussion

HT clinical presentation reflects the pathological modifications of the gland over time. After an initial diffuse swelling, the gland undergoes progressive loss of thyroid follicular cells and replacement by lymphocytes, with the formation of germinal centres and fibrosis. Apoptosis is considered to be the main pathogenetic mechanism of follicular cell destruction; a predominantly Th1-mediated immune activity may promote apoptosis of thyroid follicular cells, leading to thyroid cell destruction and HT [42], although a direct cytotoxic mechanism is also possible [43]. The gland is then colonized by B lymphocytes, which participate in the autoimmune process by inducing the formation of autoantibodies and lymphoid follicles. At the same time, the gland generally shrinks and becomes atrophic. There is no evidence about the involvement of bone marrow-derived non-haematopoietic cell types, such as endothelial progenitor cells, which have instead been associated to the pathogenesis of numerous malignancies [44-46]. Hyperplastic foci of follicular cells, accumulation of lymphoid cells, germinal centre structures and fibrosis may cause symmetrical or nodular enlargement of the gland. Complications of long standing HT include hypothyroidism and, more rarely, PTC or PTL. Despite being extremely rare, the association between HT and thyroid lymphoma has been assessed [47,48]; on the other side, the relationship between HT and PTC has been widely debated and it is still controversial [10]. Indeed, epidemiological studies highlight the coexistence of HT in 10 - 35 % of PTC patients [10,47,49]. Numerous mechanisms have been proposed to explain this association, including the expression of the RET/PTC1 and RET/PTC3 oncogenes in Hashimoto's patients [50], as well as the coexistence of similar immunohistochemical patterns, microscopic features and molecular rearrangement of the RET/PTC gene [49,51]. The clinical management of thyroid nodules, with or without HT, mainly depends on clinical data, US and FNC, the latter being the gold standard for the pre-surgical diagnosis of thyroidal nodules [40]. Despite the highest incidence of nodular thyroidal pathologies, more than 90% of the thyroid nodules are benign; therefore, FNC plays the key role to exclude surgical treatment of non-tumoral nodules. On the contrary, FNC may indicate the need for lobectomy or thyroidectomy for the neoplastic ones, on the basis of its results [18]. In this perspective, FNC plays a relevant role in the management of elderly patients in which surgical treatments are less tolerated and have additional difficulties in comparison to younger patients [32,52]. As far as HT is concerned, clinical serological and US control are sufficient for the diagnosis and clinical management in most of the cases. When HT shows nodules, masses or swelling at clinical and US evaluation, a possible neoplastic complication has to be ruled out. In these cases, FNC allows to assess the benign inflammatory nature and to exclude possible neoplastic complications. In case of cytological benign results, the indication for medical or surgical treatment is then left to the clinical and US evaluation. In case of tumoral nodules, FNC not only indicates the need for surgical treatment, but it can also be useful to draw up the following diagnostic procedures, surgery extension and possible additional treatments. In the present study, FNC proved to be a reliable diagnostic tool to confirm the clinical-cytological diagnosis of HT in its diffuse or nodular presentation. It allowed the correct medical treatment and avoided the surgical removal of nodules that would not have been properly diagnosed. Indeed, FNC correctly identified PTCs and PTLs, indicating the need and the extension of therapeutic surgical treatment.

## Conclusions

FNC has a definite role in the clinical surveillance and pre-surgical diagnosis of thyroidal diffuse enlargement and nodular presentation of HT; it can be conveniently used in elderly patients. FNC can also indicate the need for surgery and its extension in selected cases, as well as the need for medical treatments and clinical surveillance in others.

## Competing interests

The authors declare that they have no competing interests.

## Authors' contributions

PZ, MV: conception and design, interpretation of data, AC, EV, VDS, MV, AG, VDC, MPC: acquisition of data, drafting the manuscript, PZ, MV: critical revision, given final approval of the version to be published.

## Authors' information

AC = Assistant of Pathology at University of Salerno

EV = Assistant of Pathology at University of Naples "Federico II"

MV = Associate Professor of Endocrinology at University of Salerno

VDC = Aggregate Professor of Thoracic Surgery at University of Salerno

MC = Aggregate Professor of Anatomy, University of Naples "Federico II"

CC = Aggregate Professor of Oncology, University of Naples "Federico II"

AG = Aggregate Professor of Pediatric Surgery at University of Salerno

PZ = Associate Professor of Pathology at University of Salerno

## Declarations

Funding for this article has come from personal funds.

This article has been published as part of *BMC Surgery *Volume 13 Supplement 2, 2013: Proceedings from the 26th National Congress of the Italian Society of Geriatric Surgery. The full contents of the supplement are available online at http://www.biomedcentral.com/bmcsurg/supplements/13/S2
